# Biomechanical Considerations of Patching Material for Posterior Scleral Reinforcement Surgery

**DOI:** 10.3389/fmed.2022.888542

**Published:** 2022-05-16

**Authors:** Jinlei Ma, Fangyuan Wu, Zhiyong Liu, Yijiong Fang, Xu Chu, Linyan Zheng, Anquan Xue, Kaihui Nan, Jia Qu, Lingyun Cheng

**Affiliations:** ^1^School of Ophthalmology and Optometry, Wenzhou Medical University, Wenzhou, China; ^2^Eye Hospital, Wenzhou Medical University, Wenzhou, China; ^3^Jacobs Retina Center at Shiley Eye Institute, University of California, San Diego, La Jolla, La Jolla, CA, United States

**Keywords:** high myopia, posterior scleral reinforcement, human sclera, human dura mater, crosslinking, elastic modulus

## Abstract

**Purpose:**

To characterize biomechanical properties of genipin-crosslinked human dura mater as reinforcing material for posterior scleral reinforcement (PSR) and to compare it with crosslinked human sclera.

**Methods:**

Donor dura mater and sclera were crosslinked in the same optimized genipin solution. Resistance to enzyme degradation for both materials were investigated by exposing the materials to accelerated enzyme degrading. Elastic modulus and tensile strength were measured by biomechanics testing equipment. Crosslinked human dura mater was used as reinforcing patch in PSR on 57 adult pathologic myopic eyes. The patients were followed up for an average 3 years. The main outcome was eye globe axial length change and safety profile of the reinforcing material.

**Results:**

Crosslinked dura mater demonstrated similar percentage weight loss to crosslinked sclera when exposed to enzymatic solution. Dura mater has higher density than sclera. The retaining elastic modulus after enzyme exposure was 72.02 MPa for crosslinked dura mater while 53.88 MPa for crosslinked sclera, 34% greater for crosslinked dura mater, *P* = 0.0186). At the end of 3 years follow-up, the mean globe axis of the surgery eyes was reduced by 1.29 mm (from 30.81 to 29.51 mm, *P* < 0.0001, paired *t*-test). Visual acuity (BCVA logMar) improved by 0.10 logMar unit which is an improvement of five letters (*P* = 0.0184, paired *t*-test). No material specific complication was noted.

**Conclusion:**

Crosslinked human dura mater may be superior to crosslinked human sclera as reinforcing material for PSR to manage progression of high myopia. This material was well tolerated on human eye.

## Introduction

Myopia has become a worldwide health issue which is experiencing rapid worsening in recent years. In 2010, about 28% of the world's population was inflicted with myopia and that number is predicted to reach 50% by 2050 (1). Of those myopia patients, high myopia (refractive error >6 diopters) has high risk to develop myopic maculopathy and become visually handicapped in their later life. It is estimated that there were 163 million of high myopic population at year 2000 and that number will reach 938 million by 2050 (2). In some countries such as China and Japan the leading cause of blindness and low vision is not from commonly perceived age-related macular degeneration or glaucoma but from myopic maculopathy (3–5). For mild and moderate myopia, there are recent advances in controlling of the axial elongation and refractive progression, including low-concentration atropine eye drops (6, 7) and refractive measures such as orthokeratology (8, 9) and defocus contact lens (10). However, such pharmacological or optical intervention is not effective for progressive high myopia. Myopic maculopathy usually develops in fifty or later age; therefore, there are decades preceding the maculopathy. This wide time-window provides opportunities to intervene to slowdown axial elongation; unfortunately, there is no approved medical intervention available.

In recent years, posterior scleral reinforcement (PSR) surgeries have been revived to cope with fast elongation of highly myopic eyes (11). These studies have reported different surgical effect sizes and safety profiles (12–14). In general, these scleral reinforcement surgeries seem to be more or less effective on controlling rapid axial elongation (11); and the PSR procedures seem to be well tolerated (15). In contrast to the number of studies on PSR efficacy, few study investigated the effect of reinforcing material on PSR (16). We reasoned that the patching material will subject to body enzyme degrading once implanted. The tensile strength of the patch would reduce over time due to degradation and may negatively impact on efficacy of PSR. We believe that patching material is crucial for the performance of PSR. We previously demonstrated that crosslinked human sclera was superior to non-crosslinked human sclera in resistance to enzyme degradation and subsequent mechanical strength (16). Thus far, human donor sclera has been the mainstay for reinforcing material used for PSR. Human sclera alike, dura mater is also a tough and dense membrane which offers a much greater area per donor for sampling of reinforcing strips. We hypothesize that crosslinked human dura mater might be superior to crosslinked human sclera as patching material for PSR. The aim of the current study was to investigate enzymatic degrading profiles and retaining biomechanical strength of these two crosslinked fibrous membranes.

## Materials and Methods

### Optimizing and Standardizing of Crosslinking Condition

In the previous clinical PSR, human scleral patching was crosslinked in a solution of 0.1% genipin and 37.5% ethanol at 25° degree for at least 5 weeks. For the Ex Vivo study, we wanted to test the other crosslinking protocols in addition to the one we used for clinical practice because the protocol takes at least 5 weeks and easy to subject to variations due a large time-window. In addition, the termination of crosslinking was difficult to be exactly 5 weeks (840 h) and is often time with a couple of hours of variation due to busy schedules of clinical staff. It is crucial to control exact crosslinking condition in order to scientifically compare enzymology and mechanics of human sclera vs. human dura mater for the current study. Based on literature, a higher genipin and ethanol concentrations may expedite the process of crosslinking (17) to shorten the processing time (18). Due to healthy donor tissue shortage, we decided to use rabbit sclera as a model to standardize crosslinking condition for the purpose of comparison.

Ten rabbits were humanly sacrificed according to statement for the use of animals in ophthalmic and vision research. The enucleated eyes were carefully trimmed for extra ocular tissues from the scleral surface before two longitudinal scleral strips (3 mm by 15 mm) were cut out from each side of the optic nerve head. Total 40 scleral strips were acquired and randomly divided into five crosslinking conditions: (A). 0.45% genipin and 75% ethanol at 37°C for 72 h; (B). 0.45% genipin and 75% ethanol at 37°C for 168 h; (C). 0.1% genipin and 37.5% ethanol at 25°C for 5 weeks; (D). 0.6% genipin and 85% ethanol at 37°C for 72 h; (E). 0.6% genipin and 85% ethanol at 37°C for 168 h. Once the crosslinking was completed, the strips were kept in 75% ethanol within an air-tight jar for further 72 h before the mechanical testing.

### Mechanical Tensile Strength Testing

Crosslinked rabbit sclera strips (3 by 15 mm strips) under above five conditions were vertically fixed along the long axis by the tensile testing fixtures (Model: CMT2503; Mattes, Jinan). The samples were pulled vertically with a uniaxial stretching 200-N force sensor at a speed of 1 mm/min.

### Enzymatic Degradation of Crosslinked Human Sclera and Human Dura Mate

Human sclera was obtained from a local eye bank after the cornea was used for transplantation. The donor had negative serology for HIV, hepatitis B or C, and syphilis. After the donor sclera was cleaned under a surgical microscope by removing episcleral tissue, a complete evisceration was performed. The sclera shell was completely washed in distilled water and 0.9% saline before being immersed in 75% ethanol for preservation. Dura mater was acquired within 12 h after the death of donor *via* local Red Cross Organization. The dura mater was harvested by trained personnel following sterile procedure. Similar serology tests and sample cleaning were conducted as described above for scleral preparation.

Donor sclera shells or dura mater patch was soaked in 0.45% genipin and 75% ethanol at 37°C for 72 h for crosslinking. Five-millimeter diameter discs were punched out of the crosslinked donor sclera or dura mater. Thickness of the discs were measured by a ratchet force measuring device with a resolution of 0.01 mm (Shanghai Hengliang Measuring Tools Co., Ltd., Shanghai, China). Ten discs each were allocated into two groups: enzymatic solution (six discs) and PBS (four discs). The discs were incubated at 37°C for repeated cycles of 24 h. At the end of each 24-h cycle, the discs were blotted by a cotton cutip prior to drying under 37°C for 6 h. Dried discs were weighted in a precision microgram scale before entering a new cycle. Enzymatic solution was prepared by dissolving protease XIV (Streptomyces griseus Sigma Prod. No. P5147; Sigma-Aldrich, St. Louis, MO, USA) into PBS at 1 U/ml with 1% penicillin-streptomycin.

### Tensile Strength Testing of Crosslinked Human Sclera and Dura Mater Following Enzymatic Treatment

Twenty 3 by 15 mm crosslinked human scleral strips and 20 crosslinked dura mater strips were each subject to either enzymatic solution (10 strips) or PBS (10 strips) for consecutive 11 days before subsequent testing for mechanical tensile strength as described above.

### PSR With Crosslinked Human Dura Mater on Pathologic Myopic Eyes

There were 33 patients, of which 57 eyes had PSR surgery with crosslinked dura as reinforcing material; the other nine eyes had no PSR. Human donor dura mater was crosslinked in 0.1% genipin and 37.5% ethanol at 25°C for 5 weeks. All the surgery eyes had myopic macular degeneration including macular retinoschisis or macular hole (no detachment). The study adhered to the tenets of the Declaration of Helsinki and were approved by the Ethics Committee of the Eye Hospital, Wenzhou Medical University. Benefits and risks of PSR were well explained and patient's consent was obtained before the surgery. All patient eyes were subjected to the comprehensive ophthalmic exams. Ocular refractive parameters including cornea refractive power and axis, spherical refractive power, cylindrical power and axis, globe axial length by IOLMASTER (Carl Zeiss Meditec AG, Jena, Germany) were recorded prior to the surgery and the last follow-up. PSR surgical procedure was as previously reported (14) and all surgeries were performed by the same surgeon.

### Statistical Analysis

For *ex vivo* study data, means along with standard deviations or standard errors were presented. If the data are not normally distributed, comparison each mean with overall mean was performed using analysis of means by transformed ranks. To compare modulus from multiple strain locations between two types of materials, modulus was pooled and regression analysis was performed while adjusting for strain variation. Otherwise, two-sample *t*-test was used. For clinical patients' data, demographic characteristics of the patients were tabulated with means ± standard deviations for continuous data such as age, BCVA, IOP, spherical equivalent, and eye globe axial length while using counts or fractions for discrete data such as gender, grouping. The changes of IOP, visual acuity, axial length between baseline and the last follow-up were assessed by paired *t*-test. JMP statistical software was used for all analysis (JMP®, Version <16>. SAS Institute Inc., Cary, NC, 1989–2007).

## Results

### Elastic Modulus of Crosslinked Rabbit Sclera Strips

**Figure 1:**
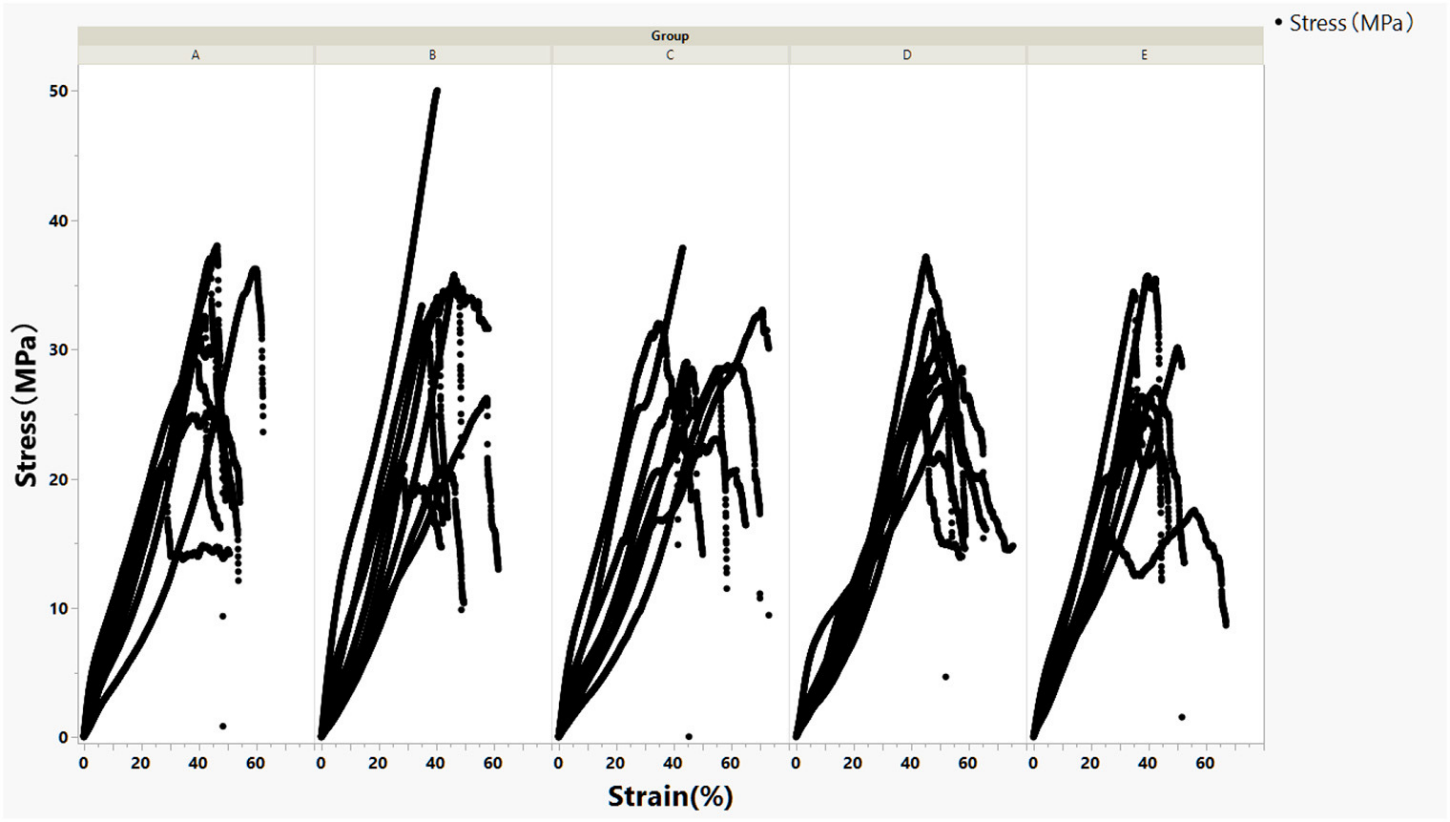
Stress–strain curves of crosslinked rabbit scleral strips stratified by five crosslinking conditions **(A–E)**.

Eight scleral strips each were crosslinked at five conditions (A–E). The stress–strain curves were demonstrated in Figure 1.

**Figure 2:**
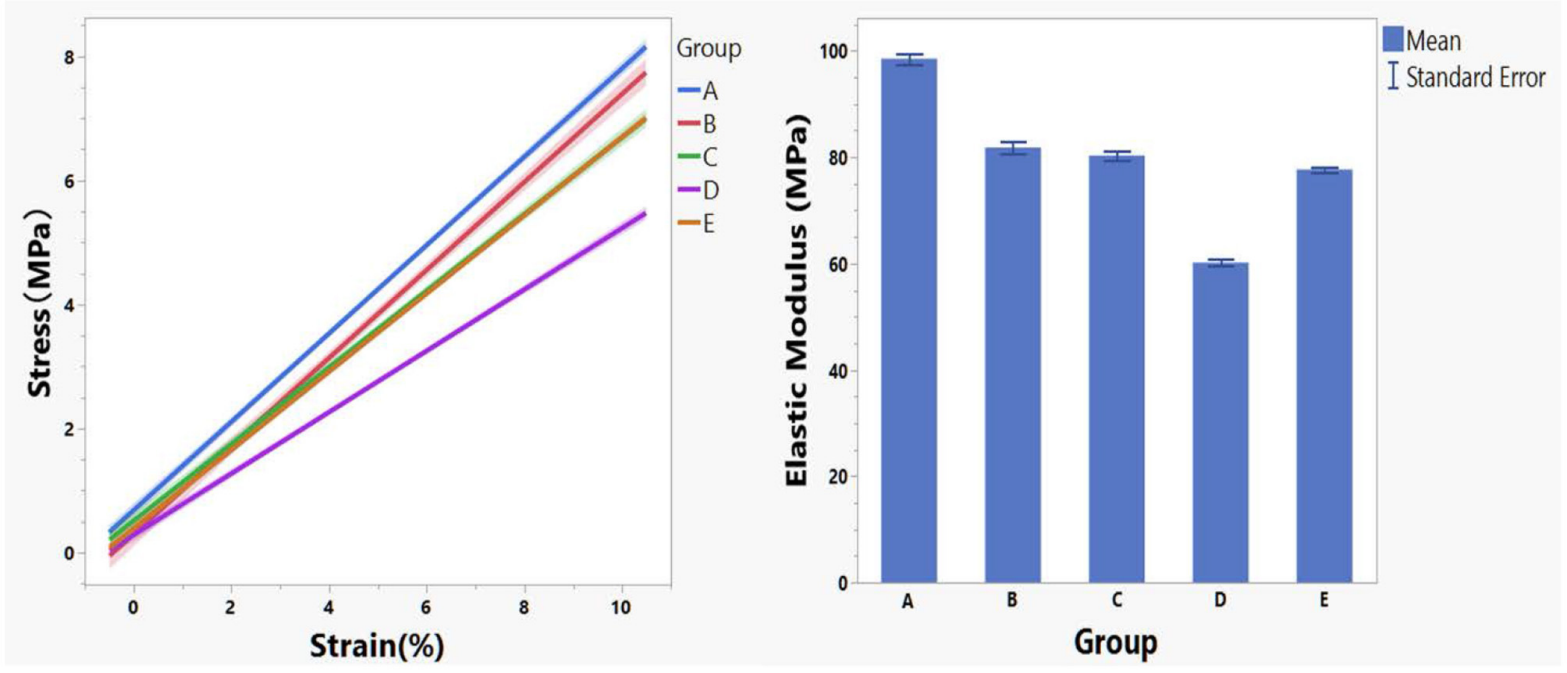
Regression lines of stress–strain data within 10% strain stratified by crosslinking conditions (left panel) and the corresponding mean modulus (right panel).

For the current study, the modulus at lower strain percent is more relevant because stretch of reinforcing patch during surgery or after implantation is very limited. The regression lines of stress–strain curves for each crosslinking condition within 10% strain and their corresponding modulus were presented in Figure 2.

Modulus data by group is not normally distributed and analysis of means by transformed ranks revealed mean modulus of group A (98.51 MPa) is significantly greater (*P* < 0.0001) than the overall mean (79.71 MPa) while modulus of group D (60.19 MPa) is significantly smaller (*P* < 0.0001) than the overall mean (79.71 MPa).

### Enzyme Degrading Profiles of Crosslinked Human Sclera and Human Dura Mater Discs

**Figure 3:**
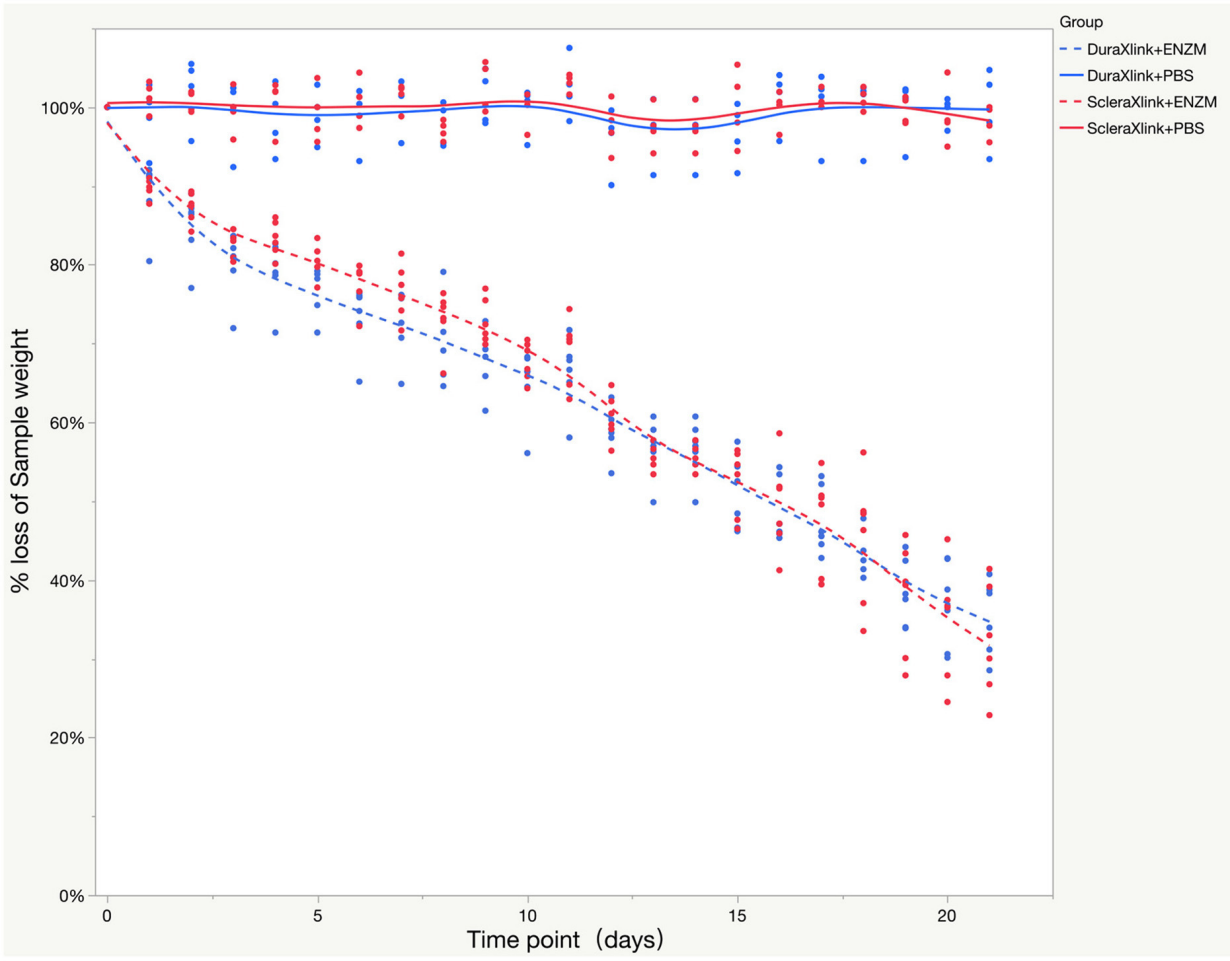
Enzyme degradation test of both crosslinked sclera and crosslinked dura mater over time while PBS was used as control. The data were presented as a percentage weight loss from original sample during 3 weeks of study. Xlink, crosslink; PBS, phosphate buffered saline; ENZM, protease XIV, 1 U/ml with 1% penicillin-streptomycin.

Human sclera and dura mater were crosslinked in 0.45% genipin and 75% ethanol at 37°C for 72 h before punching for 5-mm discs. Ten discs were randomly punched out from crosslinked sclera or dura mater. The mean weight of dura mater discs was 4.11 ± 0.51 mg vs. 3.46 ± 0.24 mg for sclera discs (*P* = 0.0031, *t*-test). The mean thickness of dura mater discs was 0.23 ± 0.043 mm vs. 0.29 ± 0.048 mm for sclera discs (*P* = 0.0003, *t*-test). Six discs were subject to enzyme degradation while 4 discs to PBS as concurrent controls. Enzyme accelerated weight loss of the discs over time; percentage weight loss was similar for both crosslinked sclera and crosslinked dura mater (Figure 3). However, remaining mean weight of the dura discs was 1.40 ± 0.31 mg while 1.16 ± 0.27 mg for sclera discs by end of the degradation study.

### Mechanical Tensile Strength of Crosslinked Sclera and Dura Strips After Enzyme Treatment

**Figure 4:**
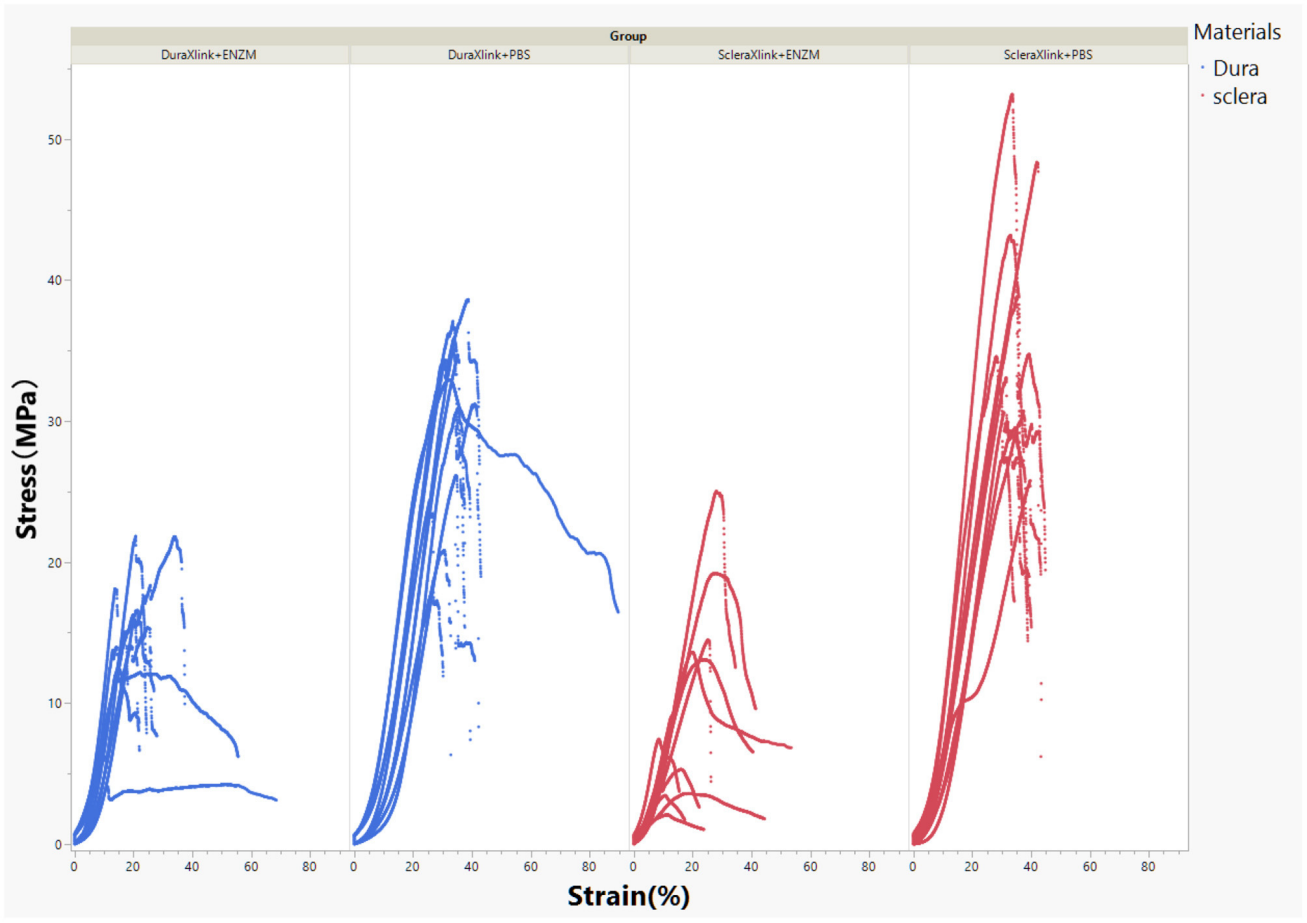
Stress–strain curves of crosslinked human sclera or dura after 11 days of exposure to enzymatic solution or to PBS. DuraXlink, crosslinked dura; ScleraXlink, crosslinked sclera; PBS, phosphate buffered saline; ENZM, protease XIV, 1 U/ml with 1% penicillin-streptomycin.

After consecutive 11 days degradation in enzymatic solution or PBS, crosslinked sclera or dura mater strips were subject to mechanical tensile strength testing. The stress–strain curves were demonstrated in Figure 4.

**Figure 5:**
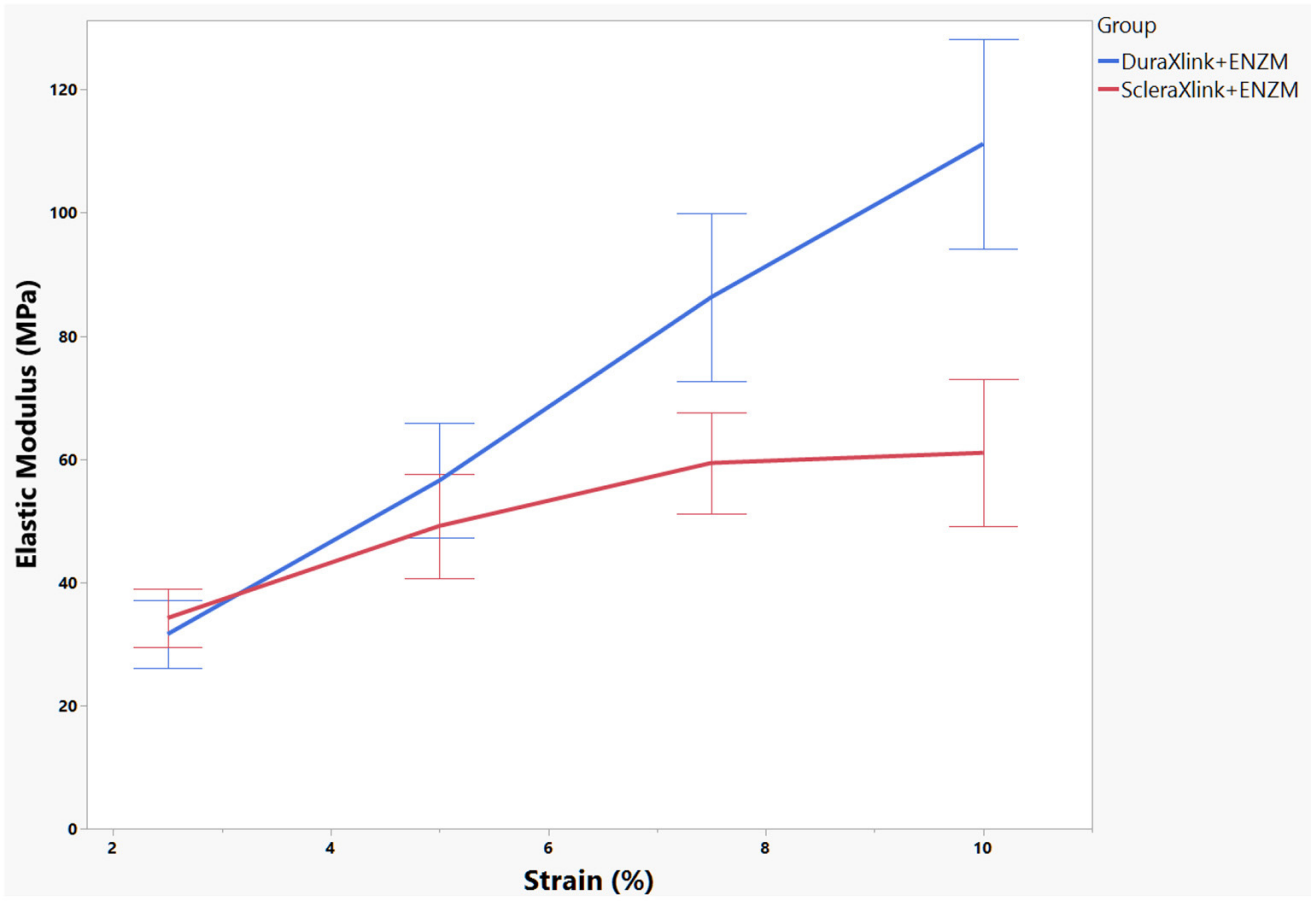
Elastic modulus of each sample was calculated at four strain locations. The mean and standard error of the modulus were presented. DuraXlink, crosslinked dura, ScleraXlink, crosslinked sclera; PBS, phosphate buffered saline; ENZM, protease XIV, 1 U/ml with 1% penicillin-streptomycin.

In general, enzyme degradation rendered both crosslinked sclera and crosslinked dura mater more rigid because the maximum loading force decreased significantly from 27.75 ± 4.34 to 11.66 ± 3.89 N for dura strips (*P* < 0.0001, Student's *t* each pair) and from 32.11 ± 4.48 to 8.94 ± 5.64 N for scleral strips (*P* < 0.0001, Student's *t* each pair). After enzyme exposure, crosslinked dura strips had significantly more tensile strength than the crosslinked scleral strips (11.66 ± 3.89 N vs. 8.94 ± 5.64 N, *P* = 0.0095, Student's *t* each pair). As the Figure 4 demonstrated, the stress–strain relationship of the crosslinked dura or sclera was nonlinear. There is a clear toe covering roughly 10% strain. In fact, modulus at this area is the most relevant to study reinforcing material used for PSR. Elastic modulus at the strains of 2.5, 5, 7.5, and 10% were presented in Figure 5. The pooled mean elastic modulus of crosslinked dura strips is significantly greater than that of crosslinked scleral strips (least square means 72.02 MPa vs. 53.88 MPa, *P* = 0.0186). If expressed as modulus of rigidity (*G*) based on formula *G* = *E*/(2^*^[1 + *v*]), elastic modulus (*E*) of 72.02 MPa is equivalent modulus of rigidity of 24.83 MPa using Poisson's ratio (*v*) as 0.45 (19); or modulus of rigidity of 18.20 MPa for an elastic modulus of 53.88 MPa using Poisson's ratio as 0.48 (20).

### SEM (Scanning Electron Microscope) Surface Profile of Crosslinked Scleral and Dura Strips After Degradation

**Figure 6:**
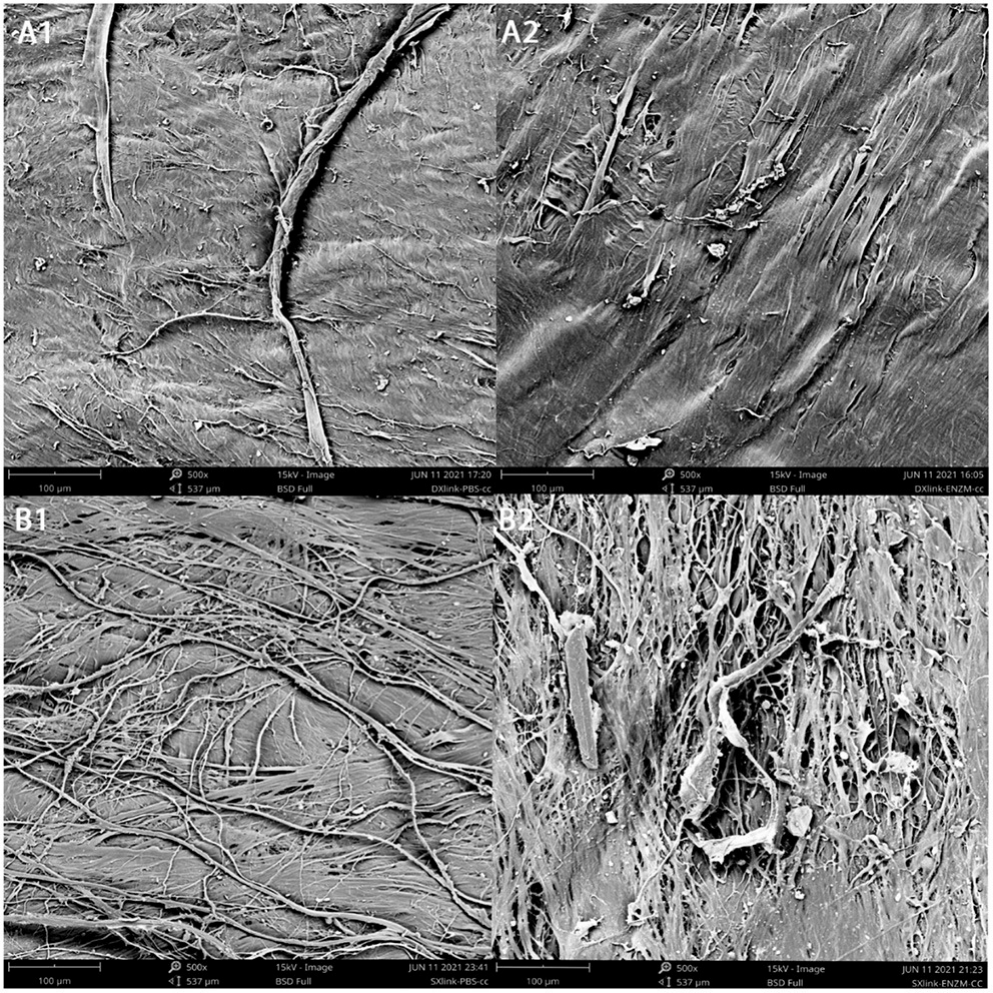
Surface appearance of concave side of the samples under SEM following degradation. **(A1)**, crosslinked dura mater in PBS; **(A2)**, crosslinked dura mater in enzyme solution; **(B1)**, crosslinked sclera in PBS; **(B2)**, crosslinked sclera in enzyme solution.

Similarly prepared and crosslinked scleral or dura strips (two strips each, one in enzyme and the other in PBS) were imaged by SEM after 11 days of degradation (Figure 6). Compared with the samples in PBS (Figures 6A1,B1), the samples in enzymatic solution (Figures 6A2,B2) showed fewer surface details such as fine running striae. Comparing surface of crosslinked dura (Figure 6A2) with crosslinked sclera (Figure 6B2) after enzyme exposure, crosslinked sclera had a rougher surface (Table 1).

**Table 1:** Surface roughness of crosslinked dura mater and crosslinked sclera after degradation.

	**Rq**	**Ra**	**Rsk**	**Rku**
DuraXlink in PBS	198.513	182.928	1.159	1.399
DuraXlin in ENZM	194.187	175.849	1.2	1.503
ScleraXlink in PBS	196.593	180.819	1.165	1.415
ScleraXlink in ENZM	203.492	189.331	1.132	1.327

*PBS, phosphate buffered saline; ENZM, enzyme solution; Rq, root mean square deviation; Ra, arithmetical mean deviation; Rsk, skewness of the assessed profile; Rku, kurtosis of the assessed profile*.

### PSR With Crosslinked Dura Mater on Adult Degenerative Myopia

The key baseline characteristics and parameters at the last follow-up were summarized within Table 2.

**Table 2:** Key baseline and last follow-up parameters of the patients and eyes.

**Age (year) at surgery**	**Gender (M/F)**	**eyes #**	**BCVA logMar**	**IOP (mmHg)**	**Spherical equivalent (diopter)**	**AL (mm)**	**Last follow-up BCVA logMar**	**Last follow-up IOP (mmHg)**	**Last follow-up AL (mm)**
48.03 ± 14.06	10/23	57 (PSR)	0.66 ± 0.39	14.30 ± 2.44	−16.80 ± 6.02	30.81 ± 1.93	0.57 ± 0.35	12.63 ± 2.88	29.51 ± 1.91
49.33 ± 10.69	4/5	9 (No PSR)	0.34 ± 0.33	12.79 ± 2.61	−4.54 ± 4.82	27.99 ± 2.91	0.27 ± 0.23	12.62 ± 3.90	27.63 ± 2.67

*BCVA, best corrected visual acuity; LogMAR, logarithm of the minimum angle of resolution; IOP, intraocular pressure; AL, axial length of the globe*.

The patients were followed-up for 2–4 years with a mean of 3.12 ± 0.88 years after the surgeries. By the end of follow-up, the mean globe axis of the surgery eyes reduced by 1.29 mm (*P* < 0.0001, paired *t*-test). Visual acuity (BCVA logMar) improved by 0.10 logMar unit which is an improvement of five letters (*P* = 0.0184, paired *t*-test). The spherical equivalents between pre- and post-surgery were not comparable because it is common for patients over 40 to seek lens refractive surgery within 1 year after retinoschisis was corrected by PSR. In fact, for those highly myopic eyes the key parameter is ocular axial length; the refractive powers of the lens and cornea contribute minimally.

As a safety profile measure, mean IOP was pairwise examined and 1.67 mmHg IOP decrease was noted (*P* < 0.0001, paired *t*-test). In addition, cylindrical refractive power was also compared between baseline and the last follow-up (−1.88 ± 1.46 diopter vs. −1.68 ± 0.93 diopter, *P* = 0.145, paired *t*-test). No surgery related safety issue was noted or reported upon the last follow-up.

## Discussion

It has been long known that highly myopic globe has a thinner (21) and weaker (22) sclera that has been attributed to progressive globe elongation. Therefore, to strengthen sclera (16, 23) has been considered as one possible management to slow myopic axial elongation. Different materials have been used for PSR, donor sclera (12, 14, 24, 25) then dura mater (13) have been used for PSR as reinforcing patches due to their good biological compatibility with the eye globe and sufficient tensile strength. We hypothesized that a patching material with higher resistance to enzyme degradation and larger retaining mechanical strength would enhance PSR performance. This hypothesis was based on our previous work in which crosslinked sclera patch achieved significant greater PSR surgical effect size than non-crosslinked scleral patch. Current study was focusing on comparison between crosslinked dura mater and crosslinked sclera. There is no verified optimal crosslinking parameter for human dura mater. We performed an *ex vivo* study using rabbit sclera as samples to find a relative optimal crosslinking condition by which we can fairly compare mechanical properties between similarly crosslinked donor sclera and dura mater. It is true that the ideal situation would be to compare the best crosslinked sclera with the best crosslinked dura for their mechanical properties. However, the best crosslinking condition is application specific and not available. To find the best crosslinking condition is out scope of the current study.

Both crosslinked dura and crosslinked sclera demonstrated similar percentage weight loss to enzyme degradation; however, crosslinked dura discs had higher initial mean weight (4.11 mg vs. 3.46 mg, *P* = 0.0031). Though similar percentage weight loss to enzyme, the remaining mass is greater for crosslinked dura mater. The greater mass may translate to stronger tensile strength. Indeed, resultant average elastic modulus of crosslinked dura strips was 26% greater than that of crosslinked scleral strips. This finding may be explained by higher collagen density of dura: same diameter discs (5 mm) but smaller thickness (0.23 vs. 0.29 mm) and greater weight (4.11 vs. 3.46 mg) for dura mater. These parameters depicted that 4.11 mg was distributed in 4.51 mm^3^ [(*r*^2^• π) • Height] of dura vs. 3.46 mg was in 5.69 mm^3^ of sclera. Clearly, dura has higher mass density. Another interesting point to elaborate is that the stress–strain relationship of the crosslinked dura or sclera was nonlinear. There is a clear toe covering roughly 10% strain area. Even within this range, nonlinearity was also clear. Therefore, elastic modulus was reported at 4 different strain percentages. The stretching magnitude of patching strip from surgery maneuver is most likely fall in this range. Nonlinearity of the stress-stretch curves has been reported in literature for natural human dura (19) and sclera (26). In the current study, SEM images depicted more changes of surface roughness for dura (Rq Δ = 6.9) than for sclera (Rq Δ = 4.32) after exposure to enzyme. It seems uneven degrading creates fissures in the sclera sample, which may be due to lower density of the scleral sample because lower density facilitate easier penetration by enzyme solution. These deep fissures may damage deeper layer collagen fibers and resultant reduced elastic modulus.

In the current study, for the first time we used genipin-crosslinked human dura mater as patching material for PSR on 57 pathologic myopic eyes. There was no concurrent PSR control using crosslinked human scleral patch; however, such a pilot study can still give us a sense of material safety and PSR efficacy. In addition, current PSR with crosslinked dura was implicated as a treatment measure instead of being a preventative measure as if on young highly myopic eyes (13, 14). The current patient cohort consists of adult highly myopic eyes which had myopic degenerative changes such as posterior staphyloma, retinoschisis, and macular hole (no detachment). It is surprising for us to see that the PSR with crosslinked dura patch completely arrested the globe axial elongation judged at 3 years after the procedure. In fact, the mean axial length was reduced by 1.29 mm. This reduction most likely came immediately with the PSR procedure when the reinforcing patch forcing outpouching posterior staphyloma inward. The globe axis stayed shorter than baseline for 3 years, which suggests that crosslinked dura patch may be strong to enzyme degrading and retaining good tensile strength along the course. This finding is encouraging. In addition, crosslinked dura mater has as good safety profile as crosslinked sclera after 3 years of follow-up (16). Originally PSR was developed and reported to slow accelerated eye growth in high myopia of children or young patients. In recent years, PSR is increasingly being used as a treatment for pathologic myopic changes such as foveoschisis and myopic macular detachment (12, 25, 27, 28). Two reports used crosslinked human sclera as reinforcing patch on pathologic myopic foveoschisis and macular detachment, which revealed similar axial reduction but the sample size was small and follow-ups were 1 year or less (27, 28). B. Ward and the colleague reported a very similar cohort on whom non-crosslinked human sclera was used for their PSR procedure and their patients were followed-up for 5 years. In that study, available 26 eyes at post-surgery 3 years showed a 0.2 mm axial elongation that was statistically significant elongation from the baseline (12). To objectively assess superiority of crosslinked dura mater over crosslinked sclera as reinforcing materials for PSR, a parallel prospective study is needed and it is advisable to longitudinally compare surgical effect sizes on progressive highly myopic eyes in children as a prophylactic measure. Adult pathologic myopic eyes can be managed by PSR for foveoschisis or macular detachment; however, macular pathologies render axial length measurement less accurate. Additionally, differing from adult high myopia the progressive high myopia in children has much higher eye globe elongation rate (29), which will facilitate to reveal the winner.

Currently, PSR is the only available option for progressive highly myopic eyes and this procedure is increasingly utilized in recent years. We acknowledge that more studies tailored for optimizing crosslinking of dura mater are needed before switching from the current crosslinking protocol that has being used for crosslinking sclera for our PSR clinical practice. This crosslinking condition has been attested by its good safety profile and medical benefit (16, 27). Though previous study was focusing on safety and efficacy of the crosslinked human sclera, the good ocular safety after 3 years of follow-ups hints that this crosslinking protocol may produce good ocular safety profile if used for crosslinking human dura mater. Ocular safety profile of the patching materials, either human donor sclera (14) or human donor dura mater (13), is known to be safe for PSR procedure. What needs to be investigated for ocular safety is the crosslinked human sclera and crosslinked human dura mater, or simply put, crosslinking agents and agents' concentrations. It has been reported that widely used crosslinking agent, glutaraldehyde, has more cytotoxicity than genipin (30). *In vivo* experimental study of skip wound healing, glutaraldehyde-crosslinked gelatin as wound covering cause more local inflammation compared with genipin-crosslinked gelatin membrane (31). Based on this information, we used “0.1% genipin and 37.5% ethanol at 25°C for 5 weeks” to crosslink dura mater for PSR on patients in the current study. As we expected, the current study did demonstrate good ocular safety profile for such crosslinked human dura mater with 3 years of follow-up.

In summary, a new reinforcing material for PSR, genipin-crosslinked human dura mater, was introduced to the high myopia research community. We demonstrated significant higher elastic modulus or rigidity of crosslinked dura mater following simulated enzyme exposure when compared with crosslinked sclera under a well-controlled experimental setting. Crosslinked dura mater as a reinforcing material patch seems to be well tolerated in PSR on myopic eyes. Current study is the first report to use crosslinked human dura mater as patching material in PSR; more related studies are welcomed to further characterize this reinforcing material. In addition, controlled longitudinal studies are warranted to objectively assess this patching material in young progressive highly myopic eyes as a prophylactic strategy.

## Data Availability Statement

The raw data supporting the conclusions of this article will be made available by the authors, without undue reservation.

## Ethics Statement

The studies involving human participants were reviewed and approved by the Ethics Committee of the Eye Hospital, Wenzhou Medical University. The patients/participants provided their written informed consent to participate in this study. The animal study was reviewed and approved by the Ethics Committee of the Eye Hospital, Wenzhou Medical University.

## Author Contributions

AX and JM equally contributed to this work. AX performed the surgeries and supervised the clinical data collection. JM along with FW, ZL, and YF performed *in vitro* and *ex vivo* studies to characterize the mechanical properties of the study materials. LZ and XC assisted AX with the surgeries and performed patient follow-ups along with clinical data collection. KN supervised material experiments and provided funding. JQ provided funding and approved the study. LC designed study, performed data analysis and interpretation, and critical revision of the manuscript. All authors contribute to writing of the manuscript.

## Funding

This work was supported by Wenzhou Major Science and Technological Innovation Project (ZY2021018), Eye Hospital, Wenzhou Medical University (YNZD2201901), and State Key Laboratory of Ophthalmology, Optometry and Vision Science, Wenzhou Medical University (J02–20190202).

## Conflict of Interest

The authors declare that the research was conducted in the absence of any commercial or financial relationships that could be construed as a potential conflict of interest.

## Publisher's Note

All claims expressed in this article are solely those of the authors and do not necessarily represent those of their affiliated organizations, or those of the publisher, the editors and the reviewers. Any product that may be evaluated in this article, or claim that may be made by its manufacturer, is not guaranteed or endorsed by the publisher.

## Abbreviations

PSR: posterior scleral reinforcement
BCVA: best corrected visual acuity
PBS: phosphate buffered saline
ENZM: enzyme solution
Rq: root mean square deviation
Ra: arithmetical mean deviation
Rsk: skewness of the assessed profile
Rku: kurtosis of the assessed profile
Xlink: crosslink
IOP: intraocular pressure
AL: axial length
SEM: scanning electron microscope.
